# Prognostic Association of TERC, TERT Gene Polymorphism, and Leukocyte Telomere Length in Acute Heart Failure: A Prospective Study

**DOI:** 10.3389/fendo.2021.650922

**Published:** 2021-03-08

**Authors:** Yanxiu Li, Iokfai Cheang, Zhongwen Zhang, Wenming Yao, Yanli Zhou, Haifeng Zhang, Yun Liu, Xiangrong Zuo, Xinli Li, Quan Cao

**Affiliations:** ^1^ Department of Critical Care Medicine, The First Affiliated Hospital of Nanjing Medical University, Nanjing, China; ^2^ Department of Cardiology, The First Affiliated Hospital of Nanjing Medical University, Nanjing, China; ^3^ Department of General Surgery, The Affiliated Jiangning Hospital of Nanjing Medical University, Nanjing, China

**Keywords:** telomere—genetics, telomerase reverse transcriptase (TERT), genetic polymorphism, heart failure, prognosis, telomerase RNA component (TERC)

## Abstract

**Background:**

Telomere length and telomerase are associated in development of cardiovascular diseases. Study aims to investigate the associations of TERC and TERT gene polymorphism and leukocyte telomere length (LTL) in the prognosis of acute heart failure (AHF).

**Methods:**

Total 322 patients with AHF were enrolled and divided into death and survival group according to all-cause mortality within 18 months. Seven single nucleotide polymorphisms (SNPs) of TERC and TERT were selected. Baseline characteristics, genotype distribution and polymorphic allele frequency, and genetic model were initially analyzed. Genotypes and the LTL were determined for further analysis.

**Results:**

Compared to carrying homozygous wild genotype, the risk of death in patients with mutated alleles of four SNPs- rs12696304(G>C), rs10936599(T>C), rs1317082(G>A), and rs10936601(T>C) of TERC were significantly higher. The dominant models of above were independently associated with mortality. In recessive models, rs10936599 and rs1317082 of TERC, rs7726159 of TERT were independently associated with long-term mortality. Further analysis showed, in haplotype consisting with TERC - rs12696304, rs10936599, rs1317082, and rs10936601, mutant alleles CCAC and wild alleles GTGT were significant difference between groups (P<0.05). CCAC is a risk factor and GTGT is a protective factor for AHF patients. Relative LTL decreased over age, but showed no difference between groups and genotypes.

**Conclusions:**

The SNPs of TERC and TERT are associated with the prognosis of AHF, and are the independent risk factors for predicting 18-month mortality in AHF.

## Introduction

Heart failure (HF) is a series of symptoms and signs caused by structural and/or functional abnormalities of the heart. Acute heart failure (AHF) is defined as a rapid onset of new or worsening of HF, which often a potentially life-threatening condition requiring immediate assessment and treatment (1). The incidence of heart failure is closely related to age and affected by the environment and the interaction of multiple genes (2). Cardiovascular disease accounted for more than 40% of the deaths for residents (3) despite the treatment advancement. Screening of high-risk patients would significantly reduce the mortality rate of patients with acute heart failure and also save medical expenses.

Aging, inflammatory response and oxidative stress are the main endogenous factors causing changes in telomere shortening and telomerase activity. Telomere shortening is associated with cardiovascular risk factors such as age, gender, smoking, sedentary lifestyle, obesity, excessive drinking, and psychological stress (4–6). In addition, atherosclerosis, essential hypertension, heart failure, coronary heart disease and other cardiovascular diseases are also accompanied by changes in telomere length and telomerase activity (7–10).

Telomeric DNA sequences of the same species are highly conserved. The length of telomere DNA gradually shortens with aging and cell division, which as a circadian clock and eventually initiate apoptosis (11, 12). Among, leukocyte telomere length (LTL) has been recognized as a clinical indicator for measuring the risk of age-related diseases. Animal studies have shown that telomere depletion is associated with apoptosis in cardiomyocytes and chronic heart failure (CHF) (13, 14). A follow-up study in CHF patients showed the shorter telomere length was associated with higher mortality and rehospitalization rate as well (15).

Genome-wide association studies (GWAS) (16–18) have demonstrated that differences in telomere length (TL) between individuals may be associated with single nucleotide polymorphisms (SNPs), of which five loci are involved in telomere biology, including chromosomes 3q26.2 (TERC), 5p15.3 (TERT), 4q32.2 (NAF1), 10q24.33 (OBFC1), 18 and 20q13.3 (RTEL1).

Telomerase is a ribonucleoprotein polymerase and composed of the telomerase reverse transcriptase (TERT), the telomerase RNA component (TERC), and the TERC-binding protein dyskerin, which plays a key role in the regulation of telomere length (TL). Changes in TL and telomerase activity are the potential pathological features of the above age-related conditions. In such pathological conditions, TERT and TERC are considered to participate in abnormally enhanced local or systemic oxidative stress of the telomere erosion and attrition. However, there is lack of study regarding the prognostic effect of TL in the acute setting of heart failure.

This study aims to further explore the polymorphisms of telomerase gene TERC and TERT, in the leukocyte telomere length regulation and the relation with the prognosis of acute heart failure.

## Methods and Materials

### Participants

This study prospectively enrolled 322 patients whom hospitalized for AHF in Cardiology Department of the First Affiliated Hospital with Nanjing Medical University from March 2012 to April 2016. Inclusion criteria were age ≥18 years, with new-onset AHF or acute exacerbation of chronic heart failure. All patients received standard treatment after admission according to the guideline (19). Patients with malignant tumor, severe mental illness, and/or uncontrolled systemic disease were excluded. The study protocols were approved by the independent Ethics Committee (First Affiliated Hospital of Nanjing Medical University, Nanjing, Jiangsu, China). Each participant had signed informed consent. The trial was registered at http://www.chictr.org.cn/(Trial registration: ChiCTR—ONC-12001944, Registered 5 Feb 2012, http://www.chictr.org.cn/showprojen.aspx?proj=7604).

### Data Collection and Follow-Up

Within 24 h admitted to the hospital, all patients underwent comprehensive clinical evaluation included demographics, physical examination, laboratory results, clinical data, medical history and etiology of AHF.

All venous blood samples were obtained at the admission or in the following morning and analyzed in the central laboratory of our hospital to measure the complete blood count and other biochemical markers. Transthoracic echocardiography (TTE) was used for evaluating the left ventricular systolic and diastolic function on the Vivid E9 ultrasound system (GE Medical System, United States of America).

The primary endpoint was all-cause mortality during the 18-month follow up. Patients were evaluated for the primary endpoint by out-patient visit, telephone evaluation, and/or confirmation of their family or physician every 3 months. Patients were separated into survival group and death group according to the primary endpoint.

### Single Nucleotide Polymorphism Selection

The genes were selected using the genome database of Chinese Han Beijing (CHB) population and review of previous relative studies literature related to association between TERC and TERT polymorphisms (20–26). 7 SNPs were selected in for further analysis including TERC (rs12696304, rs10936599, rs1317082, rs10936601, rs16847897) and TERT(rs7726159, rs2736100). The minor allele frequencies (MAF) of all the selected SNPs were greater than 5%.

### Genotyping and Leukocyte Telomere Length Measurement

Genomic DNA was extracted from whole blood samples using the TIANamp Blood DNA Kit (DP318; TIANGEN, Beijing, China) and the concentration was measured by spectrometry (NanoDrop 2000 spectrophotometer, Thermo Scientific, Waltham, MA, USA).

TaqMan fluorescent probe quantitative PCR technology were used for the SNP genotyping. Based on the nucleic acid sequences of the seven selected SNPs, the allele-specific TaqMan probes were designed, synthesized and verified by Thermo Fisher Scientific. Reaction system included TaqPath ProAmp Master Mix 2.5 μl, Assay Mix Probe [20×] 0.25 μl, Genomic DNA/Nuclease-Free Water (ddH2O) 2.25 μl (5 ng/ul). Genotyping were performed using ABI PRISM 7900HT Sequence Detection System 2.4 (SDS2.4) in accordance with the manufacturer’s protocol.

Genotyping quality control procedures leading to SNP exclusion were call rate <90% and P<0.05 for deviations from Hardy-Weinberg equilibrium (HWE). The selected SNPs in the study were successfully genotyped with 99.68% of call rate. With 36B4 for internal control and ETV6 as primer, each LTL sample was measured using a multiplex quantitative real-time PCR method and was calculated by T/S ratios (T, telomere signal; S, single copy gene signal) (27–29).

### Statistical Analysis

Continuous variables were expressed as the mean ± standard deviation (SD) and compared by student’s t-test or one-way ANOVA for normal distribution, or expressed as median with inter-quartile range (IQR) and compared by Mann-Whitney U test or Kruskal-Wallis H test for skewed distribution. Categorical variables and frequency of events were reported as numbers (percentages) and compared by chi-square test. In the correlation analysis, after the logarithmic transformation of the data that skewed distribution, the Pearson method is used for the analysis. The online SHESIS software (http://analysis.bio-x.cn/myAnalysis.php) was used to analyze the HWE, genotype, allele frequency distribution, linkage disequilibrium and SNP haplotypes (30, 31). Kaplan-Meier and multi-variable COX analysis was used to analyze the prognosis of AHF patients under different genetic models of SNPs. Correlation analysis between haplotype and AHF prognosis was performed, the P value was subjected to FDR (False Discovery Rate) correction and Bonferroni correction.

All statistical analyses were two-sided and the significance level was set to P < 0.05. When D’ >0.8 and r^2^ >0.33, linkage disequilibrium (LD) was considered between sites. SPSS version 19.0 statistical package (SPSS, Chicago, IL, USA) and Microsoft Excel were used for all statistical analyses.

## Results

### Baseline Characteristics

In total, 322 AHF patients were divided into death group (80 cases) and survival group (242 cases). The detail characteristics of the participants between two groups were shown in **Table 1**. There were 15 variables from the baseline characteristics (**Table 1**) were considered to be statistically significant. Variables included systolic and diastolic blood pressure, aspartate aminotransferase (AST), albumin (ALB), Serum creatinine (Scr), blood urea nitrogen (BUN), uric acid (UA), CystatinC (CysC), serum potassium (K), serum sodium (Na), hemoglobin (HB), D-Dimer, NT-proBNP, pulmonary artery systolic pressure (PASP), and comorbidities of renal dysfunction (All P<0.05). In addition, there was no significant difference in sex distribution, treatment regimen, NYHA classification, and other comorbidities (All P>0.05).

**Table 1:** Baseline characteristics between survival group and death group.

Characteristic	Totals (n=322)	Survivals (n=242)	Deaths (n=80)	*P*
Age (year-old)	60.51 ± 16.24	59.62 ± 16.41	63.21 ± 15.50	0.086^a^
Sex (F/M)	110/212	76/166	34/46	0.078^b^
BMI (Kg/m2)	24.27 ± 4.53	24.27 ± 4.50	24.26 ± 4.65	0.982^a^
HR (bpm)	86 ± 22	87 ± 22	85 ± 22	0.411^a^
SBP (mmHg)	125 ± 22	128 ± 23	118 ± 17	0.000^a^
DBP (mmHg)	78 ± 15	80 ± 16	74 ± 12	0.000^a^
ALT (U/L)	26.70 (17.73, 46.68)	31.30 (21.80, 49.70)	24.25 (15.83, 58.13)	0.165^c^
AST (U/L)	29.30 (22.90, 44.18)	30.70 (24.00, 43.90)	27.40 (21.70, 46.35)	0.034^c^
ALB (g/L)	37.36 ± 4.67	37.71 ± 4.67	36.27 ± 4.52	0.020^a^
Scr (umol/L)	87.10 (72.05, 109.95)	89.60 (76.00, 110.70)	96.95 (78.63, 137.88)	0.004^c^
BUN (mmol/L)	7.19 (5.82, 9.54)	7.28 (6.17, 8.71)	8.49 (6.14, 10.90)	0.000^c^
UA (mmol/L)	473.0 (382.0, 582.0)	479.0 (388.0, 576.0)	526.5 (446.0, 748.0)	0.029^c^
CysC (mg/L)	1.31 (1.12, 1.63)	1.31 (1.10, 1.57)	1.52 (1.23, 1.77)	0.001^c^
K (mmol/L)	3.99 ± 0.51	3.96 ± 0.48	4.09 ± 0.56	0.042^a^
Na (mmol/L)	139.76 ± 4.09	140.19 ± 3.81	138.49 ± 4.62	0.004^a^
Ca (mmol/L)	2.25 ± 0.14	2.25 ± 0.14	2.25 ± 0.14	0.993^a^
HB (g/L)	133.08 ± 20.81	135.24 ± 20.08	126.55 ± 21.70	0.001^a^
RDW (%)	14.95 ± 4.78	14.75 ± 5.35	15.54 ± 2.29	0.205^a^
D-dimer (mg/L)	0.75 (0.30, 1.72)	0.59 (0.29, 1.58)	0.94 (0.31, 2.84)	0.001^c^
NT-proBNP (ng/L)	1979 (1176, 4315)	1775 (1205, 2933)	2626 (1688, 6615)	0.000^c^
cTnT (ng/L)	61.80 ± 387.34	45.82 ± 295.11	101.74 ± 556.69	0.380^a^
CK-MB (U/L)	35.34 ± 122.90	36.27 ± 139.96	32.98 ± 61.23	0.869^a^
TTE				
LVDd (mm)	61.53 ± 12.51	61.35 ± 11.96	62.08 ± 14.12	0.661^a^
LVDs (mm)	48.85 ± 14.01	48.67 ± 13.52	49.39 ± 15.53	0.697^a^
PASP (mmHg)	42.0 (31.5, 53.0)	40.0 (31.0, 48.0)	48.0 (30.5, 60.5)	0.005^c^
LVEF%	42.50 ± 14.81	42.43 ± 14.57	42.71 ± 15.62	0.885^a^
**NYHA**				0.228^b^
II (n%)	48 (14.9)	40 (16.5)	8 (10.0)	
III (n%)	181 (56.2)	137 (56.6)	44 (55.0)	
IV (n%)	93 (28.9)	65 (26.9)	28 (35.0)	
**Comorbidities**				
IHD (n%)	76 (23.6)	55 (22.7)	21 (26.3)	0.545^b^
Cardiomyopathy (n%)	130 (40.4)	101 (41.7)	29 (36.3)	0.431^b^
VHD (n%)	86 (26.7)	63 (26.0)	23 (28.8)	0.663^b^
PHD (n%)	23 (7.1)	17 (7.0)	6 (7.5)	1.000^b^
Atrial fibrillation (n%)	122 (37.9)	94 (38.8)	28 (35.0)	0.596^b^
CHD (n%)	10 (3.1)	6 (2.5)	4 (5.0)	0.273^b^
HTN (n%)	162 (50.3)	125 (51.7)	37 (46.3)	0.440^b^
DM (n%)	77 (23.9)	55 (22.7)	22 (27.5)	0.450^b^
Pulmonary Infection (n%)	66 (20.5)	48 (19.8)	18 (22.5)	0.633^b^
Renal dysfunction (n%)	20 (6.2)	10 (4.1)	10 (12.5)	0.013^b^
Thyroid Dysfunction				0.763^b^
Hyperthyroidism(n%)	7 (2.2)	6 (2.5)	1 (1.3)	
Hypothyroidism(n%)	1 (0.3)	1 (0.4)	0 (0)	
Smoking (n%)	121 (37.6)	98 (40.5)	23 (28.8)	0.064^b^

F, Female; M, Male; BMI, Body Mass Index; HR, Heart Rate; SBP, Systolic Blood Pressure; DBP, Diastolic Blood Pressure; ALT, Alanine Aminotransferase; AST, Aspartate Aminotransferase; ALB, Albumin; Scr, Serum Creatinine; BUN, Blood Urea Nitrogen; UA, Uric Acid; CysC, CystatinC; HB, Hemoglobin; RDW, Red blood cell Distribution Width; NT-proBNP, N-terminal prohormone of brain natriuretic peptide; cTnT, cardiac troponin T; CK-MB, Creatine kinase-MB; LVDd, Left Ventricular Diastolic Dimension; LVDs, Left Ventricular Systolic Dimension; PASP, Pulmonary Artery Systolic Pressure; LVEF, Left Ventricular Ejection Fraction; IHD, Ischemic Heart Disease; VHD, Valvular Heart Disease; PHD, Pulmonary Heart Disease; CHD; Congenital Heart Disease; HTN, Hypertension; DM, Diabetes Mellitus.

^a^Calculated by unpaired t-test; ^b^calculated by Chi-square test; ^c^calculated by rank sum test.

### Distribution of the Genotypes and Allele Frequencies

The distribution of genotypes and allele frequency (**Table 2**) of the seven SNPs of TERC and TERT genes were consistent with HWE in the death group and survival group of patients with acute heart failure (P>0.05), indicating sample has a population representative.

**Table 2:** Distribution of the genotypes and allele frequencies.

Gene	SNPs	Group	Genotype					HWE		Allele		HR (95%CI)	χ2	P
			MM	Mm	mm	χ^2^	P	χ^2^	P	M	m			
TERC	rs12696304	D	26(0.329)	41(0.519)	12(0.152)	10.00	0.0068	0.41	0.5244	93(0.589)	65(0.411)	1.81 (1.24–2.63)	9.75	0.0018
	G/C	S	128(0.529)	93(0.384)	21(0.087)			0.48	0.4875	349(0.721)	135(0.279)			
	rs10936599	D	13(0.163)	44(0.550)	23(0.287)	12.17	0.0023	1.1	0.2934	70(0.438)	90(0.562)	1.87 (1.31–2.69)	11.77	0.0006
	T/C	S	86(0.355)	115(0.475)	41(0.169)			0.06	0.8089	287(0.593)	197(0.407)			
	rs1317082	D	13(0.163)	44(0.550)	23(0.287)	13.10	0.0014	1.1	0.2934	70(0.438)	90(0.562)	1.92 (1.34–2.76)	12.75	0.0004
	G/A	S	87(0.360)	116(0.479)	39(0.161)			0.00	0.9744	290(0.599)	194(0.401)			
	rs10936601	D	27(0.338)	40(0.500)	13(0.163)	9.79	0.0075	0.08	0.7775	94(0.588)	66(0.412)	1.82 (1.25–2.63)	9.99	0.0016
	T/C	S	128(0.529)	93(0.384)	21(0.087)			0.48	0.4875	349(0.721)	135(0.279)			
	rs16847897	D	26(0.325)	41(0.512)	13(0.163)	0.60	0.7411	0.22	0.6368	93(0.581)	67(0.419)	0.87 (0.61–1.26)	0.53	0.4674
	C/G	S	90(0.372)	117(0.483)	35(0.145)			0.09	0.7604	297(0.614)	187(0.386)			
TERT	rs7726159	D	27(0.338)	32(0.400)	21(0.263)	5.38	0.0679	3.06	0.0804	86(0.537)	74(0.463)	1.26 (0.88–1.81)	1.64	0.201
	C/A	S	83(0.343)	122(0.504)	37(0.153)			0.51	0.4737	288(0.595)	196(0.405)			
	rs2736100	D	24(0.300)	35(0.438)	21(0.263)	3.41	0.1815	1.23	0.2683	83(0.519)	77(0.481)	0.79 (0.55–1.13)	1.63	0.2022
	A/C	S	78(0.322)	123(0.508)	41(0.169)			0.4	0.525	279(0.576)	205(0.424)			

SNP, single nucleotide polymorphism; HWE, Hardy-Weinberg equilibrium; HR, hazard ratio; D, death group; S, survival group; M, major allele; m, minor allele.

Among which the genotype distribution and polymorphic allele frequencies of the four loci of TERC gene were statistically different between the two groups (P<0.05):

rs12696304 (G/C, Hazard Ratio - HR=1.82, 95% CI: 1.25–2.63, P=0.0016);

rs10936599 (T/C, HR=1.87, 95% CI: 1.31–2.69, P=0.0006);

rs1317082 (G/A, HR=1.92, 95% CI: 1.34–2.76, P=0.0004);

rs10936601 (T/C, HR=1.82, 95% CI: 1.25–2.63, P=0.0016);

The genotype distribution and polymorphic allele frequency of the other three SNPs (rs16847897, rs7726159, and rs2736100) showed no significance between groups (P>0.05).

### Comparison of the Single Nucleotide Polymorphism Genotype Under Different Genetic Models

Genetic model analysis showed the genotype distribution and comparison between the death and survival groups of the seven selected SNPs of TERC and TERT genes under different genetic models are shown in **Table 3**.

- Both the dominant and recessive model genotype of rs10936599 (CC+TC *vs.* TT, HR:2.84 [1.48–5.44]; CC *vs.* TT+TC, OR:1.98 [1.10–3.57]) in TERC were statistically different between the death group and the survival group (P<0.05).
- Both the dominant and recessive model genotype of rs1317082 (AA+GA *vs.* GG, HR:2.89 [1.51–5.54]; AA *vs.* GG+GA, HR:2.10 [1.16–3.80]) in TERC were statistically different between the death group and the survival group (P<0.05).
- The dominant model genotype distribution of rs12696304 (CG+CC *vs.* GG, HR:2.33 [1.37–3.97]) in TERC was statistically different between the death group and the survival group (P<0.05).
- The dominant model genotype distribution of rs10936601 (CC+TC *vs.* TT, HR:2.20 [1.30–3.74]) in TERC was statistically different between the death group and the survival group (P<0.05).
- The recessive model genotype distribution of rs7726159 (AA *vs.* CC+CA, HR:1.97 [1.07–3.63]) in TERT was statistically different between the death group and the survival group (P<0.05).

**Table 3:** Comparison of the SNP genotype under different genetic models between the death and survival groups.

Gene	SNPs	Group	Dominance		HR (95%CI)	χ2	P	Recessive		HR (95%CI)	χ2	P
			Mm + mm	MM				mm	MM + Mm			
TERC	rs12696304	D	53(0.671)	26(0.329)	2.29 (1.34–3.90)	9.53	0.002	12(0.152)	67(0.848)	1.89 (0.88–4.03)	2.74	0.133
	G/C	S	114(0.471)	128(0.529)				21(0.087)	221(0.913)			
	rs10936599	D	67(0.838)	13(0.163)	2.84 (1.48–5.44)	10.5	0.001	23(0.287)	57(0.713)	1.98 (1.10–3.57)	5.26	0.035
	T/C	S	156(0.645)	86(0.355)				41(0.169)	201(0.831)			
	rs1317082	D	67(0.838)	13(0.163)	2.89 (1.51–5.54)	10.9	0.001	23(0.287)	57(0.713)	2.10 (1.16–3.80)	6.17	0.021
	G/A	S	155(0.640)	87(0.360)				39(0.161)	203(0.839)			
	rs10936601	D	53(0.663)	27(0.338)	2.20 (1.30–3.74)	8.83	0.003	13(0.163)	67(0.838)	2.04 (0.97–4.30)	3.65	0.062
	T/C	S	114(0.471)	128(0.529)				21(0.087)	221(0.913)			
	rs16847897	D	54(0.675)	26(0.325)	1.23 (0.72–2.10)	0.57	0.503	13(0.163)	67(0.838)	1.15 (0.57–2.30)	0.15	0.718
	C/G	S	152(0.628)	90(0.372)				35(0.145)	207(0.855)			
TERT	rs7726159	D	53(0.663)	27(0.338)	1.03 (0.60–1.75)	0.01	1.000	21(0.263)	59(0.738)	1.97 (1.07–3.63)	4.89	0.03
	C/A	S	159(0.657)	83(0.343)				37(0.153)	205(0.847)			
	rs2736100	D	56(0.700)	24(0.300)	1.11 (0.64–1.92)	0.14	0.782	21(0.263)	59(0.738)	1.75 (0.96–3.18)	3.35	0.073
	A/C	S	164(0.678)	78(0.322)				41(0.169)	201(0.831)			

SNP, single nucleotide polymorphism; HR, hazard ratio; D, death group; S, survival group; M, major allele; m, minor allele.

The major/minor alleles were G/C (rs12696304), T/C (rs10936599), G/A (rs1317082), T/C (rs10936601), C/G (rs7726159), C/A (rs1317082) and A/C (rs2736100) respectively.

### Association Between Genetic Polymorphisms and Prognosis

**Figure 1:**
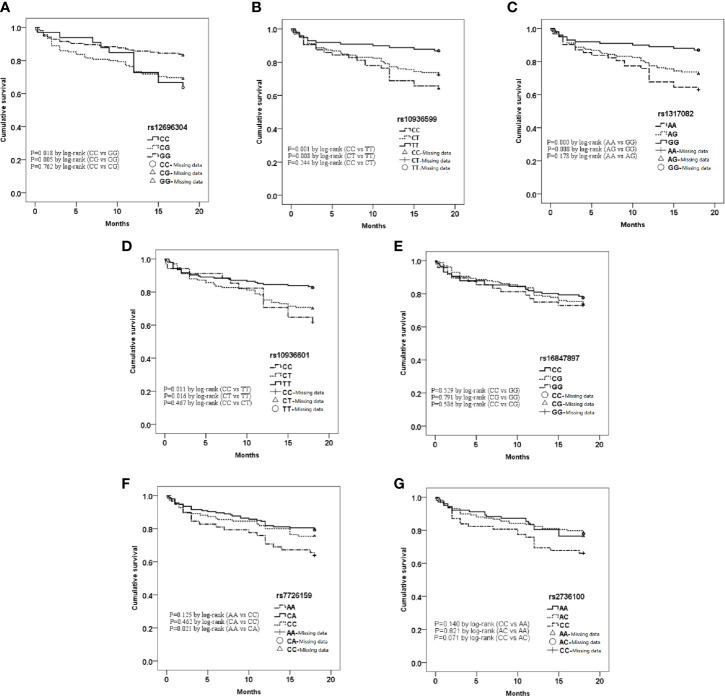
Kaplan-Meier curve analysis of 7 single nucleotide polymorphisms (SNPs) in different genotypes. **(A)** rs12696304; **(B)** rs10936599; **(C)** rs1317082; **(D)** rs10936601; **(E)** rs16847897; **(F)** rs7726159; **(G)** rs2736100.

To further assess the association between each selected SNP and the prognosis of acute heart failure, Kaplan-Meier curve analysis were used (**Figures 1A–G**). Results showed that the overall survival rate decreased over time.

- For rs12696304, rs10936599, rs1317082, and rs10936601 of TERC gene, the survival rate of AHF patients carrying mutant alleles were significantly lower than the homozygous wild alleles (P<0.05, **Figures 1A–D**).
- Under both dominant and recessive models, rs10936599 (**Figure 1B**) and rs1317082 (**Figure 1C**) were associated with decreased survival in patients with AHF (P<0.05);
- Dominant models of rs12696304 (**Figure 1A**) and rs10936601 (**Figure 1D**) were associated with decreased survival rate of AHF patients (P<0.05);
- Recessive model of rs7726159 (**Figure 1F**) was associated with a decrease in survival rate in patients with AHF (P<0.05).
- The other two SNPs—rs16847897 (**Figure 1E**) and rs2736100 (**Figure 1G**) did not show differences in neither the models between the death group and the survival group (P>0.05).

Afterward, univariate COX regression analysis all showed significance in the five SNPs (TERC—rs12696304, rs10936599, rs1317082, rs10936601; TERT—rs7726159). Wild genotype was set as the reference genotype. The association of different genetic models and prognosis of AHF were shown in **Table 4**.

**Table 4:** COX regression analysis of different genetic models.

	Genotype		χ^2^	HR (95%*CI*)	*P*
rs12696304	Codominance	GG	–	1.0(ref.)	–
		CC	8.16	3.35(1.46–7.66)	0.004
		CG	8.96	2.65(1.40–5.02)	0.003
	Dominance	GG	–	1.0(ref.)	–
		CC+CG	10.82	2.79(1.52–5.16)	0.001
	Recessive	GG+CG	–	1.0(ref.)	–
		CC	–	–	0.071
rs10936599	Codominance	TT	–	1.0(ref.)	–
		CC	11.1	4.98(1.94–12.79)	0.001
		TC	6.95	3.27(1.36–7.89)	0.008
	Dominance	TT	–	1.0(ref.)	–
		CC+TC	9.05	3.72(1.58–8.74)	0.003
	Recessive	TT+TC	–	1.0(ref.)	–
		CC	5.9	2.07(1.15–3.72)	0.015
rs1317082	Codominance	GG	–	1.0(ref.)	–
		AA	12.39	5.35(2.10–13.63)	0
		GA	6.17	3.05(1.27–7.37)	0.013
	Dominance	GG	–	1.0(ref.)	–
		AA+GA	8.76	3.63(1.55–8.51)	0.003
	Recessive	GG+GA	–	1.0(ref.)	–
		AA	8.35	2.35(1.32–4.18)	0.004
rs10936601	Codominance	TT	–	1.0(ref.)	–
		CC	6.58	3.07(1.30–7.23)	0.01
		TC	10.81	3.01(1.56–5.79)	0.001
	Dominance	TT	–	1.0(ref.)	–
		CC+TC	11.91	3.02(1.61–5.66)	0.001
	Recessive	TT+TC	–	1.0(ref.)	–
		CC	–	–	0.16
rs7726159	Codominance	CC	–	1.0(ref.)	–
		AA	6.82	2.69(1.28–5.65)	0.009
		CA	0.68	1.32(0.69–2.53)	0.409
	Dominance	CC	–	1.0(ref.)	–
		AA + CA	–	–	0.12
	Recessive	CC + CA	–	1.0(ref.)	–
		AA	4.47	1.96(1.05–3.65)	0.034

Adjusted with systolic and diastolic blood pressure, aspartate aminotransferase, albumin, Serum creatinine, blood urea nitrogen, uric acid, cystatinC, serum potassium, serum sodium, hemoglobin, D-Dimer, NT-proBNP, pulmonary artery systolic pressure, and comorbidity of renal dysfunction.

After adjusted with the 15 significant variables in the baseline characters, results showed that for rs12696304, rs10936599, rs1317082, and rs10936601 of TERC, the risk of death carrying mutation alleles were higher than those of wild homozygous genotypes, and remained as independent risk factors in AHF patients. The dominant models of these four SNPs were all independently associated with the risk of death in AHF patients (P>0.05).

Furthermore, the recessive models of rs10936599, rs1317082 of TERC, and rs7726159 of TERT were independently associated with the risk of death in AHF patients (P>0.05).

### Haplotype Analysis of Telomerase RNA Component and Telomerase Reverse Transcriptase Genes

**Figure 2:**
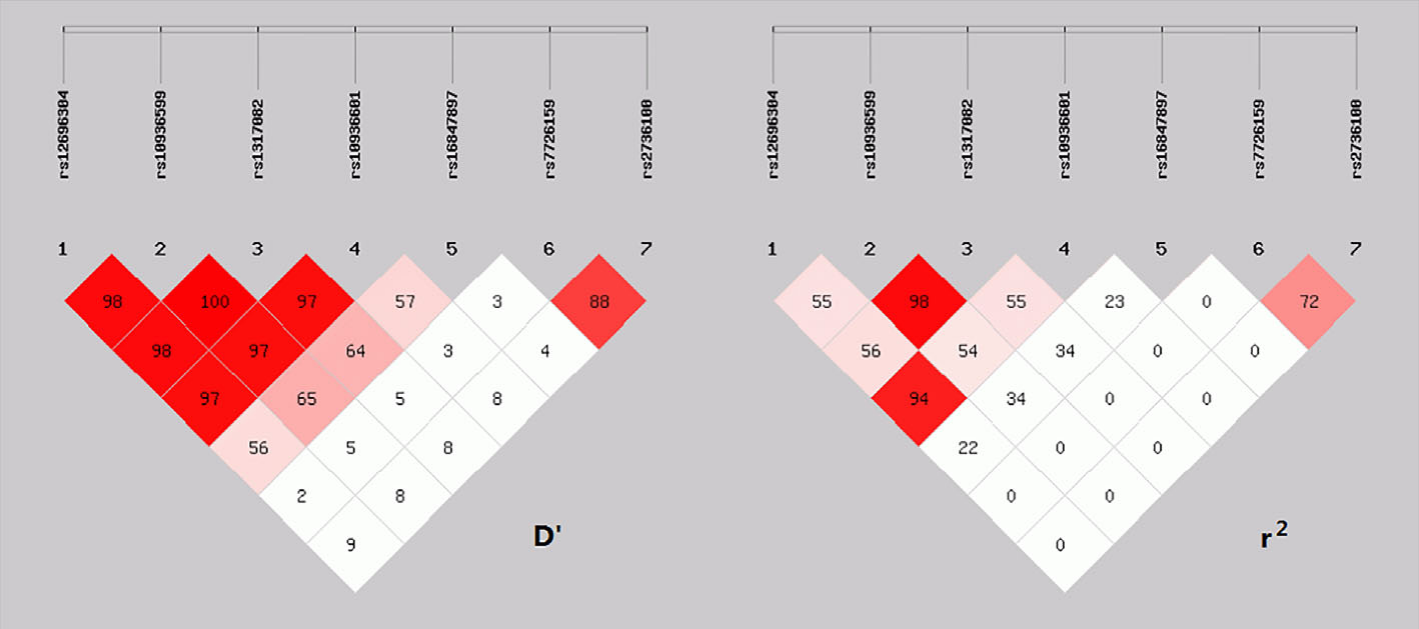
Linkage disequilibrium analysis of 7 SNPs.

The linkage disequilibrium (LD) analysis of five SNPs of TERC and two SNPs of TERT in AHF patients is shown in **Figure 2**. Further haplotype analysis was performed based on the results of linkage disequilibrium analysis.

The four haplotypes consisting of rs12696304 (G/C), rs10936599 (T/C), rs1317082 (G/A) and rs10936601 (T/C) sites, which the overall frequency was above 1%, showed statistically significant in overall haplotype distribution between the survival and death groups (P=0.0031).

The frequency of haplotype CCAC (H1) was significantly higher in the death group (OR: 1.79 [1.23–2.61], P<0.05); The frequency of haplotype GTGT (H4) was significantly lower in the death group than in the survival group (OR: 0.54 [0.38–0.78], P < 0.05). After corrected by Bonferroni or FDR method, the two haplotypes CCAC and GTGT composed of these four SNPs remained significant (P<0.05, **Table 5**).

**Table 5:** Comparison of haplotypes with TERC gene.

	Haplotypes	Combinations	Deaths(freq)	Survivals(freq)	χ^2^	P	HR (95%CI)	P_b_	P_f_
TERC	H_1_	CCAC	62.97(0.400)	132.99(0.275)	9.219	0.0024	1.79 (1.23–2.61)	0.0096	0.0096
	H_2_	CCAT	2.03(0.013)	0.75(0.002)	3.587	0.0583	8.32 (0.97–71.67)	0.2332	0.1166
	H_3_	GCAT	22.47(0.147)	59.51(0.123)	0.462	0.4969	1.20 (0.71–2.02)	1.9876	0.6625
	H_4_	GTGT	68.50(0.428)	284.48(0.588)	10.826	0.001	0.54 (0.38–0.78)	0.004	0.001
	Global P	–	158	484	11.527	0.0031	–	–	

Haplotypes were in the order of rs12696304(G/C), rs10936599(T/C), rs1317082(G/A) and rs10936601(T/C), respectively.

HR, Hazard Ratio; Pb, Bonferroni adjusted P-values; Pf, False Discovery Rate corrected P-values.

The four haplotypes consisting of the rs7726159 (C/A) and rs2736100 (A/C) of TERT gene showed no statistical difference between the groups (P>0.05, **Table 6**).

**Table 6:** Comparison of haplotypes with TERT gene.

	Haplotypes	Combinations	Deaths(freq)	Survivals(freq)	χ^2^	P	HR (95%CI)	P_b_	P_f_
TERT	H_1_	AA	7.41(0.046)	10.44(0.022)	2.73	0.0986	2.20 (0.84–5.75)	0.3944	0.3944
	H_2_	AC	66.59(0.416)	185.56(0.383)	0.543	0.4611	1.15 (0.80–1.65)	1.8444	0.9222
	H_3_	CA	75.59(0.472)	268.56(0.555)	3.284	0.07	0.72 (0.50–1.03)	0.28	0.0933
	H_4_	CC	10.41(0.065)	19.44(0.040)	1.685	0.1943	1.66 (0.77-3.61)	0.7772	0.1943
	Global P	–	160	484	6.12	0.106	–	–	

Haplotypes were in the order of rs7726159(C/A) and rs2736100(A/C), respectively.

HR, Hazard Ratio; Pb, Bonferroni adjusted P-values; Pf, False Discovery Rate corrected P-values.

### Leukocyte Telomere Length

**Figure 3:**
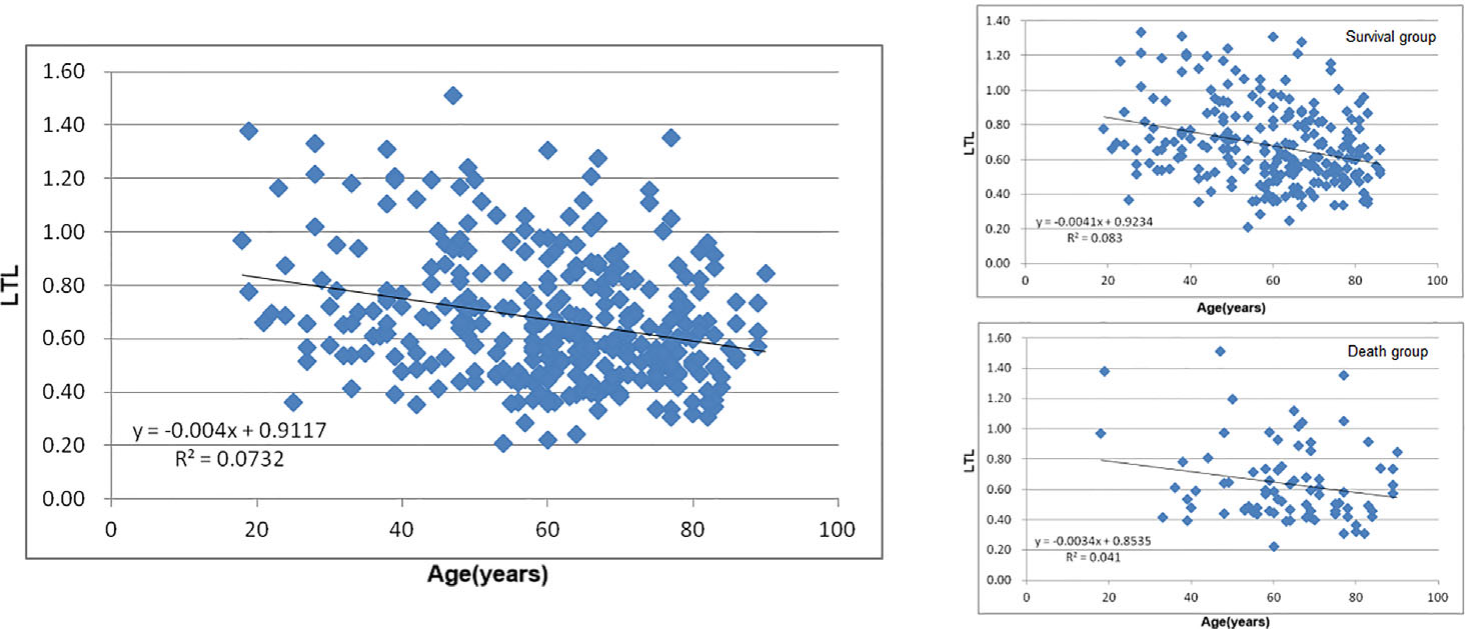
Leukocyte Telomere Length (LTL) in different age and prognosis.

Correlation analysis was performed on the difference of LTL between different prognoses and genotypes. There was a significant negative correlation between the relative LTL and age of AHF patients regardless of the primary endpoint (P<0.001, **Figure 3**), and no significant correlation with the clinical baseline (P>0.05, **Appendix 1**). Moreover, there was no significant difference had found in the research SNPs and their genotypes (P>0.05, **Appendix 2**).

## Discussion

Previous studies showed that telomerase, in addition to its nuclear-specific telomere elongation, also has extranuclear non-telomere elongation. TERT as the catalysis subunit of telomerase can regulate the level of mitochondrial reactive oxygen (32, 33). The telomere length or telomerase gene TERC and TERT polymorphisms were correlated with age, the morbidity of tumors and chronic cardiovascular diseases (34–42). These past studies were mostly regarding in chronic development diseases, and the results usually showed correlation of TL, SNPs and the morbidity of these chronic conditions. On the other hand, an underlying mechanism of decreased or stayed LT in acute settings (43). The acute stress on the heart induces compensatory mechanisms aimed at preserving TL by upregulating TERT. Despite increases in TERT, TL decreased or stayed the same in early phrase. As the heart disease progresses, however, these mechanisms become attenuated and then exhausted, leading to telomere attrition and overt cardiac failure.

To further explore the prognostic values of seven TERC and TERT genes single nucleotide polymorphisms (SNPs), and leukocyte telomere length (LTL) in AHF. By using both SNP and haplotype analysis method, we analyzed the relationship between TERC and TERT gene polymorphisms and the prognosis AHF from the perspective of epigenetics, avoiding the false negative or false positive results that might be caused by analyzing a single site.

Our results showed the genotypes rs12696304 (G>C), rs10936599 (T>C), rs1317082 (G>A), and rs10936601 (T>C) of TERC were the independent risk factors for death in AHF patients after 18 months follow-up, which suggesting these sites can be used to assess the prognosis of patients with AHF.

Haplotype analysis revealed a linkage disequilibrium between the four SNPs above (rs12696304, rs10936599, rs1317082, and rs10936601). The haplotype CCAC consisted of the mutant alleles and the haplotype GTGT consisted of the wild-type alleles of these four SNPs were significant differences between the death group and survival group. Haplotype CCAC is a risk haplotype for patients with AHF, and haplotype GTGT is a protective factor for patients with AHF.

Although the other three selected SNPs did not showed significance between groups; further analysis in the recessive model for rs7726159 of TERT were found related to the prognosis of patients as well. The results of TL analysis showed that there were no significant differences between groups in LTL regarding all genotypes of the seven SNPs in TERC and TERT.

Combined with our results of haplotype analysis, the mechanism might be different in the acute setting of disease. The biological functions and its prognostic influence might not be related directly to LTL, and may be related to its regulation mechanism besides telomere elongation of telomerase. The telomere length shortens in the effects of endogenous factors and attenuates cardiometabolism (44). In AHF, the wild genotype might provide physiological effects in protective regulation pathways by enhanced telomerase activity acting on telomeres. On the other hand, mutant genotype lost the effects and tend to be more susceptible of the endogenous factors, therefore demonstrated a higher mortality.

Although the underlying mechanisms remain to be systematically investigated, this study offers the prognostic factor of AHF from a molecular biology perspective—the effects of TERC and TERT gene polymorphisms in patients with AHF.

Within the selected SNPs, there were five SNPs of TERC and TERT genes showed a significant correlation to the prognosis of AHF. These five SNPs are all located in the non-coding region, where rs10936599 is located in the 5’UTR region, and the remaining four SNPs (rs12696304, rs1317082, rs10936601, and rs7726159) are located in the intron region. The variation of TERC or TERT gene may affect the transcription process, resulting in changing the expression level of the corresponding protein, which may eventually affect the progression of AHF.

Studies showed that LTL and adipose tissue was highly correlated (45). As smoking, sedentary lifestyle, and obesity are also factors associated with an increased burden of inflammation. Similar to TL, adipose tissue are also associated with adverse cardiometabolic risk factors, and often exhibits proinflammatory and prooxidative metabolic changes (46–48), which might associated to the direct damaging effects of adipose tissue on telomeres and the mediation through the expression of corresponding genes, such as TERT and TERC. On the other hand, adipose tissues showed various regulatory effects on cardiovascular system. Among, epicardial adipose tissue (EAT) regulates physiological and pathophysiological processes in the heart. Although not investigated in our study, our findings provide fundamental knowledge regarding TL and AHF; adipose tissue, especially epicardial adipose tissue, might be involved in these pathological mechanisms. TL and telomerase may be attributed to these regulations of metabonomics in adipocyte.

In addition, this study also has certain limitations. Firstly, the study was a single-center study with a relatively small sample size, which needs a larger cohort to further verify the correlation of the above SNPs and the prognosis of AHF patients. Secondly, the biological functions of the above positive SNPs in AHF are still unclear. Lastly, our study SNPs only included limited sites. Further researches regarding wider genome and the association with adipose tissue in heart failure are needed.

## Conclusion

The results suggest a potential association between TERC, TERT gene variants and AHF. It provided a valuable prognostic information and will better elucidate the genetic and telomeric mechanisms of patients with acute heart failure. Further genomics and lipidomics investigations are needed.

## Data Availability Statement

The original contributions presented in the study are included in the article/**Supplementary Material**. Further inquiries can be directed to the corresponding authors.

## Ethics Statement

The studies involving human participants were reviewed and approved by the Ethics Committee of the First Affiliated Hospital, Nanjing Medical University. The patients/participants provided their written informed consent to participate in this study.

## Author Contributions

YL participated in the design of the research and drafted the manuscript. IC participated in the design and is a major contributor in writing the manuscript. ZZ participated in the analyses. WY and HZ performed the analysis and interpretation of the study statistic design. YZ, YL, and XZ supervised the study program and method feasibility. QC and XL contributed to the conception and design of the research, and performed critical revision of the manuscript for important intellectual content. All authors contributed to the article and approved the submitted version.

## Funding

This study received grant support from the Twelve-Fifth National Key Technology R&D Program (2011BAI11B08) and Jiangsu Province Technology Alliance of Cardiovascular Disease (KFSN201401).

## Conflict of Interest

The authors declare that the research was conducted in the absence of any commercial or financial relationships that could be construed as a potential conflict of interest.
