# Supplementary figures and images for: Prognostic Association of TERC, TERT Gene Polymorphism, and Leukocyte Telomere Length in Acute Heart Failure: A Prospective Study
Source: Front Endocrinol (Lausanne). 2021 Mar 8;12:650922. doi: 10.3389/fendo.2021.650922 (PMC7982721; doi:10.3389/fendo.2021.650922)

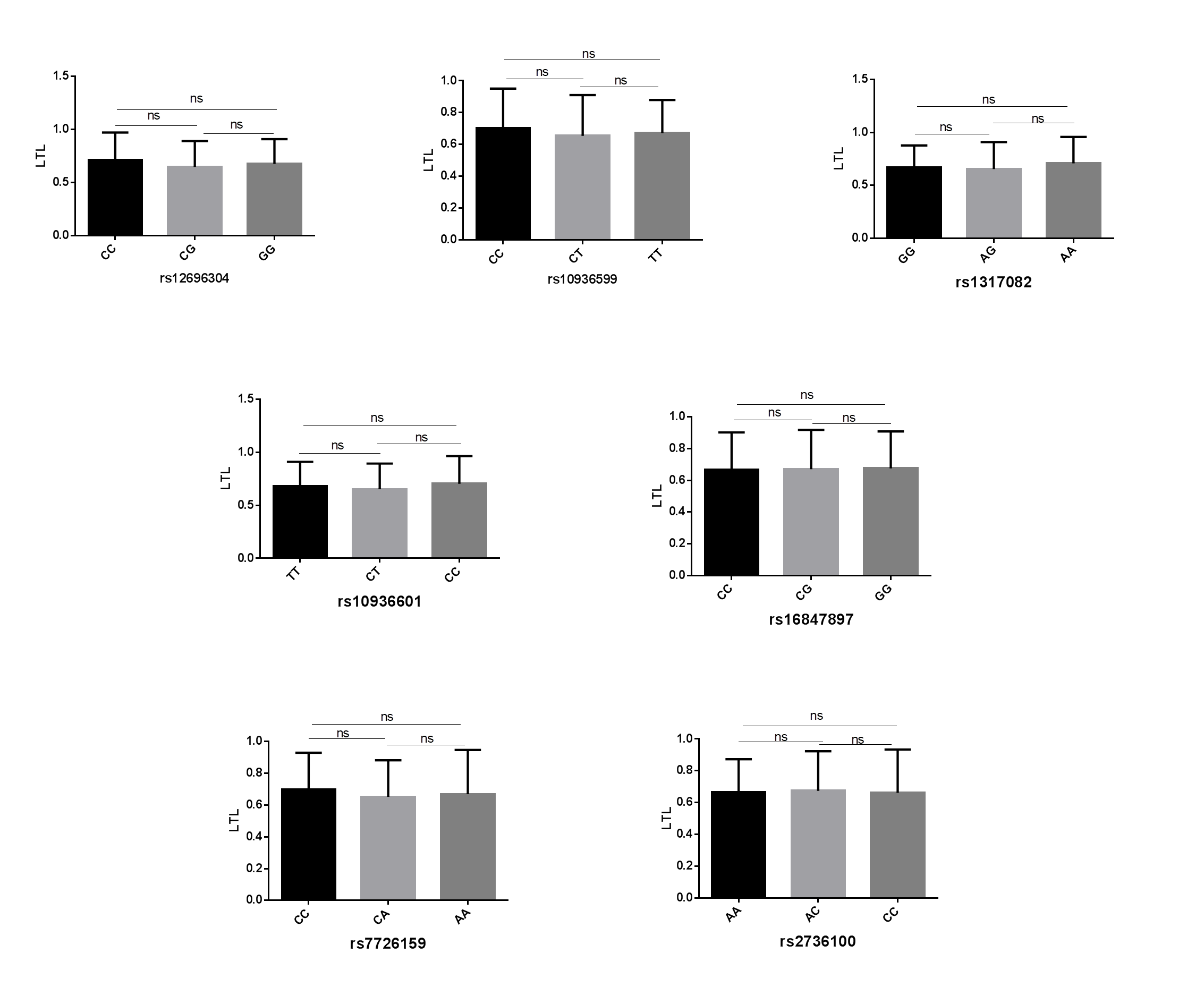

Supplement: Supplementary file 1 [file Image_1.tif]
