# Supplementary material for: Prognostic Association of TERC, TERT Gene Polymorphism, and Leukocyte Telomere Length in Acute Heart Failure: A Prospective Study
Source: Front Endocrinol (Lausanne). 2021 Mar 8;12:650922. doi: 10.3389/fendo.2021.650922 (PMC7982721; doi:10.3389/fendo.2021.650922)
Supplement: Supplementary file 2 [file Table_1.docx]

***Appendix 1 - Correlation between LTL and baseline in patients with AHF***

| Variable |  | Age | BMI | HR | SBP | DBP | K | Na | Ca |
| --- | --- | --- | --- | --- | --- | --- | --- | --- | --- |
| LTL | r | -0.270 | 0.071 | 0.001 | -0.076 | 0.000 | 0.083 | -0.097 | -0.006 |
|  | P | 0.000 | 0.263 | 0.990 | 0.174 | 0.996 | 0.140 | 0.084 | 0.912 |
|  |  |  |  |  |  |  |  |  |  |
|  |  | Ln(ALT) | Ln(AST) | ALB | Ln(Scr) | Ln(BUN) | Ln(UA) | Ln(CysC) | Ln(D-dimer) |
|  | r | 0.084 | 0.056 | 0.053 | 0.041 | -0.012 | -0.036 | 0.031 | -0.027 |
|  | P | 0.136 | 0.316 | 0.348 | 0.465 | 0.835 | 0.521 | 0.612 | 0.635 |
|  |  |  |  |  |  |  |  |  |  |
|  |  | HB | RDW | CKMB | Ln(proBNP) | LVDd | LVDs | Ln(PASP) | LVEF |
|  | r | 0.080 | -0.039 | -0.041 | -0.071 | 0.031 | 0.030 | -0.046 | -0.037 |
|  | P | 0.154 | 0.488 | 0.577 | 0.222 | 0.580 | 0.601 | 0.443 | 0.512 |

BMI:Body Mass Index; HR: Heart Rate; SBP: Systolic Blood Pressure; DBP: Diastolic Blood Pressure; K: Potassium; Na: Sodium; Ca: Calcium; ALT: Alanine Aminotransferase; AST: Aspartate Aminotransferase; ALB: Albumin; Scr: Serum Creatinine; BUN: Blood Urea Nitrogen; UA: Uric Acid; CysC: CystatinC; HB: Hemoglobin; RDW: Red blood cell Distribution Width; CK-MB: Creatine kinase-MB; NT-proBNP: N-terminal prohormone of brain natriuretic peptide; LVDd: Left Ventricular Diastolic Dimension; LVDs: Left Ventricular Systolic Dimension; PASP: Pulmonary Artery Systolic Pressure; LVEF: Left Ventricular Ejection Fraction.
